# Enteric and systemic postprandial lactate shuttle phases and dietary carbohydrate carbon flow in humans

**DOI:** 10.1038/s42255-024-00993-1

**Published:** 2024-02-22

**Authors:** Robert G. Leija, Casey C. Curl, Jose A. Arevalo, Adam D. Osmond, Justin J. Duong, Melvin J. Huie, Umesh Masharani, George A. Brooks

**Affiliations:** 1 Exercise Physiology Laboratory, Department of Integrative Biology, University of California, Berkeley, CA USA; 2 Department of Medicine, University of California, San Francisco, CA USA

**Keywords:** Dietary carbohydrates, Neuroendocrine diseases, Metabolism, Physiology

## Abstract

Dietary glucose in excess is stored in the liver in the form of glycogen. As opposed to direct conversion of glucose into glycogen, the hypothesis of the postprandial lactate shuttle (PLS) proposes that dietary glucose uptake is metabolized to lactate in the gut, thereby being transferred to the liver for glycogen storage. In the present study, we provide evidence of a PLS in young healthy men and women. Overnight fasted participants underwent an oral glucose tolerance test, and arterialized lactate concentration and rate of appearance were determined. The concentration of lactate in the blood rose before the concentration of glucose, thus providing evidence of an enteric PLS. Secondary increments in the concentration of lactate in the blood and its rate of appearance coincided with those of glucose, which indicates the presence of a larger, secondary, systemic PLS phase driven by hepatic glucose release. The present study challenges the notion that lactate production is the result of hypoxia in skeletal muscles, because our work indicates that glycolysis proceeds to lactate in fully aerobic tissues and dietary carbohydrate is processed via lactate shuttling. Our study proposes that, in humans, lactate is a major vehicle for carbohydrate carbon distribution and metabolism.

## Abstract (web summary)

This study addresses the hypothesis of dietary glucose handling in humans through a postprandial lactate shuttle.

## Main

The history of discoveries on the pathway of carbohydrate disposal can be traced to studies of isolated amphibian muscles studied without circulation or oxygenation^1–4^. Thinking around the process and regulation of glycolysis and the resulting cloud of understanding about aerobic and anaerobic processes can be traced to the same set of formative discoveries. However, few efforts have been made to understand glycolysis in the gastrointestinal tract after carbohydrate nutrition. Previously, based on rodent experimentation we posited the presence of a PLS^5^, but little experimental support was available in humans. Evidence supporting the presence of a PLS included: the ‘glucose paradox’ or ‘indirect pathway of hepatic glycogen synthesis’^6^, postprandial lactate production in rat muscle after a glucose challenge^7,8^ and portoperipheral lactate gradients in rats given an intestinal glucose load^9^. In aggregate, those results indicate lactate production in the intestinal lumen or wall and systemic lactate production after an oral glucose challenge. We sought to test for the presence of a PLS in humans using an oral glucose tolerance test (OGTT), primed continuous infusion (CI) of glucose and lactate tracers and ‘arterialized’, warmed hand vein blood sampling; the presence of enteric and systemic PLS phases is indicated.

**Fig. 1:**
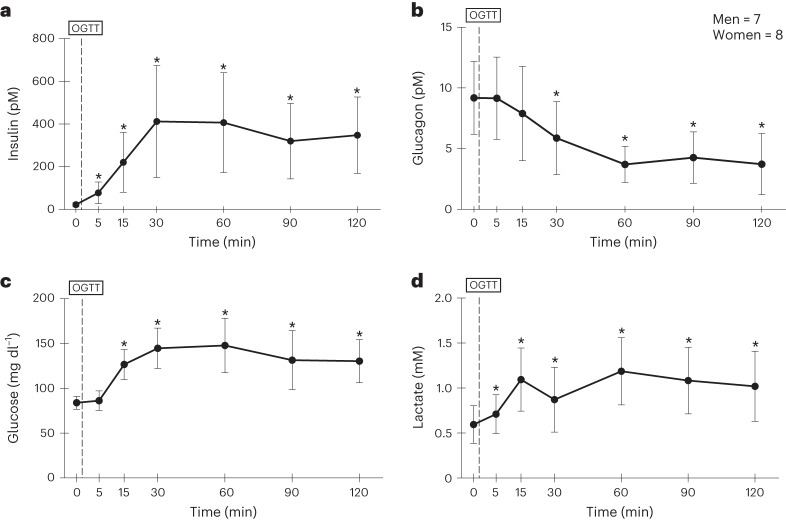
Concentrations of plasma insulin, glucagon, blood glucose and lactate in relation to the OGGT. Arterial [lactate] and insulin rise before glucose rises and glucagon falls. **a**–**d**, Plasma insulin (**a**) and glucagon (**b**), blood glucose (**c**) and lactate (**d**) concentrations before, during and after an OGTT. Values are mean ± s.d. ^*^Significantly changed from baseline, pre-OGTT levels (*P* < 0.05). A two-tailed ANOVA with corrections for repeated comparisons using Dunnett’s tests was employed. The statistical significance for group differences was determined at *α* = 0.05. Source data

Volunteers could be classified as healthy, physically active young men and women (Supplementary Table 1), but not athletes in training^10,11^. Insulin and glucagon responses are shown in Fig. 1a,b, respectively. In the figures, time zero represents an average of 75- and 90-min values after initiation of isotope tracer infusion. The 5-min and subsequent time points represent time after consumption of 75 g of d-glucose (that is, post-challenge). In the present study, we provide mean ± s.d. for the volunteers studied. Consistent with previous observations, insulin rose and glucagon fell in response to an OGTT (*P* < 0.05). It is interesting that there was a rapid, significant post-challenge rise in insulin at 5 min that preceded the fall in glucagon or the rise in arterialized blood [glucose] (*P* < 0.05).

Arterialized blood glucose and lactate concentrations are presented in Fig. 1c,d, respectively. From baseline blood [glucose] was significantly elevated at 15 min (*P* < 0.05) and peaked 60 min after the oral challenge (*P* < 0.05). In contrast, blood lactate concentrations rose from baseline within 5 min (*P* < 0.05); arterial [lactate] peaked at 15 min post-consumption (*P* < 0.05). Immediately after the spike in blood [lactate], we observed a significant decline at 30 min post-challenge (*P* < 0.001). The nadir in blood [lactate] at 30 min was followed by a steady rise above baseline with a second peak at 60 min, after which [lactate] remained elevated (*P* < 0.05).

**Extended Data Fig. 1:**
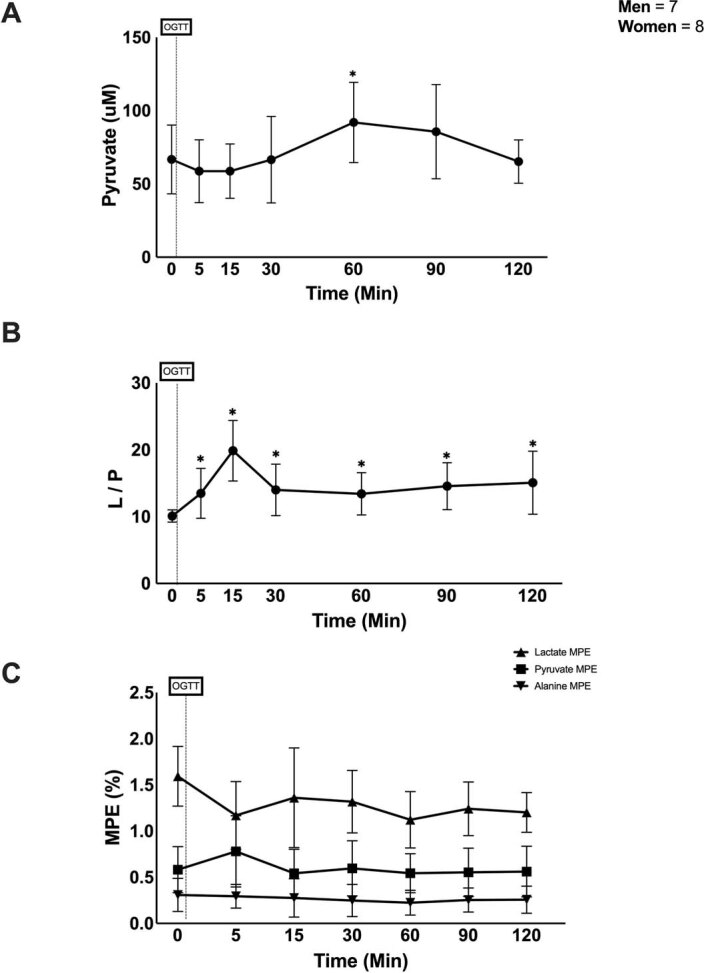
Time course of blood pyruvate concentrations (A) the lactate to pyruvate ratio (L/P) (B) and isotopic enrichments of lactate, pyruvate, and alanine (C) before, during and after an oral glucose tolerance test (OGTT). Values are means SD. *Significantly increased from baseline (*p* < 0.05). For Fig. 1a, b a two-tailed, one-way analyses of variance (ANOVA) with corrections for repeated comparisons using Dunnett’s tests. Statistical significance for group differences was determined at *α* = 0.05. Source data

The arterial lactate:pyruvate ratio (L:P) was nominally 10 before the oral glucose challenge, rose to 20 by 15 min after the glucose challenge and remained ≥10 for the duration of observations (*P* < 0.05). Changes in the L:P were mainly the result of changes in [lactate], not [pyruvate] (Extended Data Fig. 1a,b). Isotopic enrichments (IEs) of pyruvate approximated half those of lactate and IEs of alanine approximated one-fifth of lactate and did not correlate (*r* = 0.04) (Extended Data Fig. 1c).

**Fig. 2:**
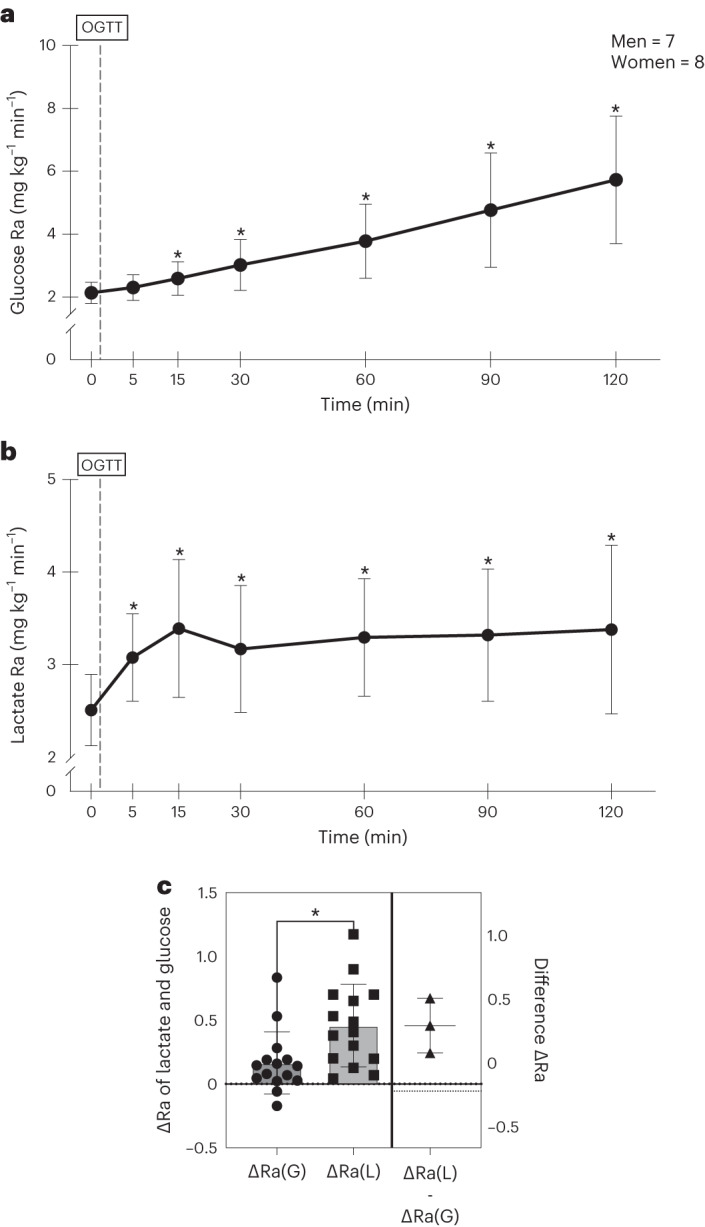
Rates of appearance of glucose and lactate in relation to the OGGT. Glucose was fed but arterial lactate appearance (Ra) rose before glucose Ra. **a**,**b**, Glucose and lactate rates of appearance (glucose Ra) (**a**) and (lactate Ra) (**b**) before, during and after an OGTT. **c**, Changes in glucose Ra and lactate Ra before and 5 min after glucose challenge. Values are mean ± s.d. ^*^Significantly increased from baseline (*P* < 0.05). For **a** and **b** a two-tailed ANOVA with corrections for repeated comparisons using Dunnett’s tests was employed and for **c** a two-tailed, unpaired Student’s *t*-test was applied. G, glucose; L, lactate. The statistical significance for group differences was determined at *α* = 0.05. Source data

There were clear differences in blood glucose and lactate kinetics post-glucose challenge. Arterial glucose rate of appearance (Ra) was significantly higher than baseline from 15 min to 120 min post-glucose challenge (Fig. 2a; *P* < 0.05). In contrast, post-challenge blood lactate Ra followed a bimodal pattern featuring an initial rise from pre-OGTT at 5 min and 15 min (Fig. 2b; *P* < 0.05). As with blood [lactate], this initial phase in the lactate Ra response was followed by a nadir at 30 min, with a subsequent steady rise in lactate Ra (*P* < 0.05). Hence, the initial peak in blood [lactate] after an oral glucose challenge (Fig. 1d) was the result of a sudden rise in blood lactate appearance (Fig. 2b). In accordance with these changes, increases in Ra from baseline to 5 min post-glucose challenge were significantly greater for lactate than for glucose (*P* < 0.05) (Fig. 2c).

**Fig. 3:**
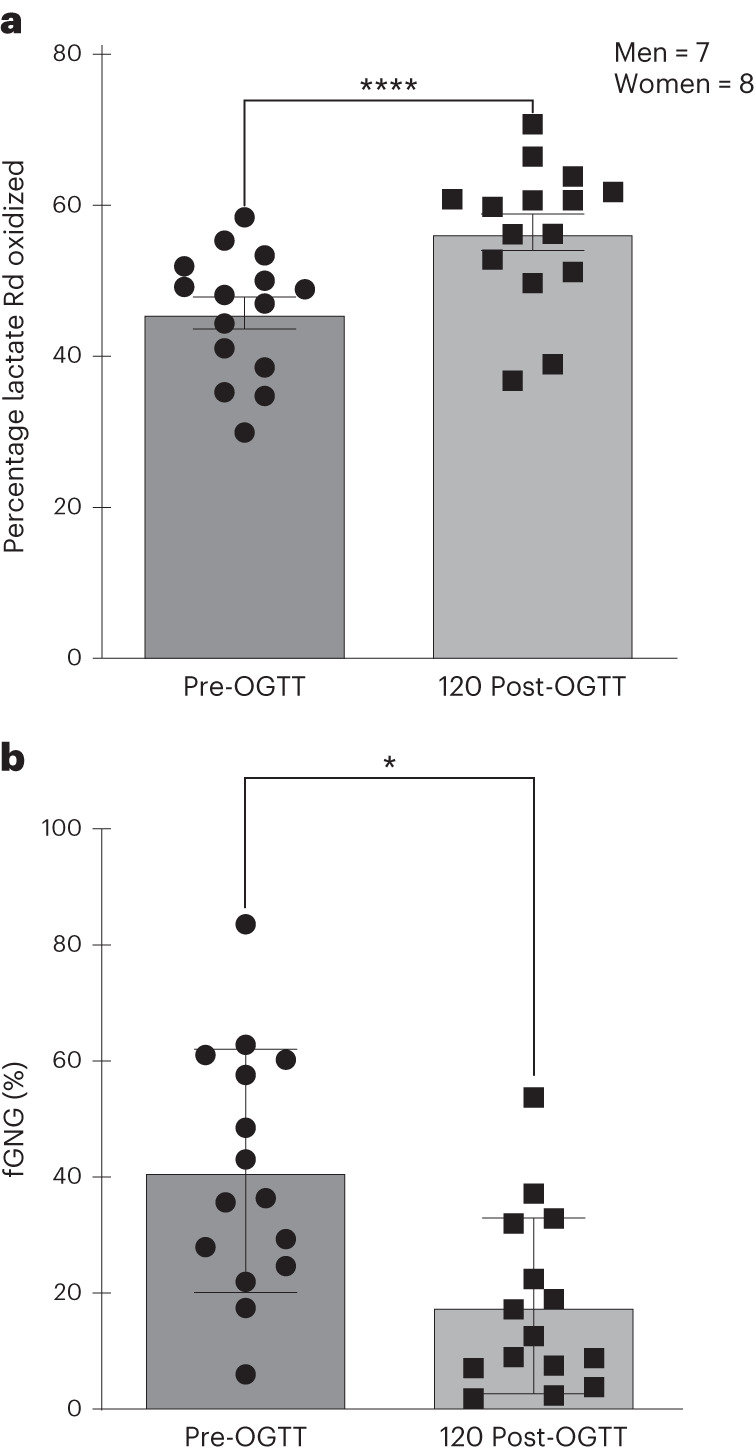
Percentage disappearance of and fractional GNG from lactate in relation to the OGGT. A shift in lactate disposal from GNG to oxidation following an OGTT. **a**,**b**, Percentage of lactate disappearance disposed of via oxidation (**a**) and fractional (f) GNG from lactate (**b**) before and after an OGTT. Values are mean ± s.d. ^*^Significantly decreased from baseline (*P* < 0.05). A two-tailed, paired Student’s *t*-test was applied. The statistical significance for group differences was determined at *α* = 0.05. Source data

Lactate oxidation rates rose significantly post-glucose challenge (Fig. 3a). The percentage of lactate disposal via oxidation increased significantly from pre-OGTT (46 ± 8%) to post-glucose challenge (57 ± 8%, *P* < 0.05). Owing to increasing systemic PLS phase glucose appearance (Fig. 2a), presumably from hepatic glucose release^12^, the percentage of lactate disposed of via gluconeogenesis (GNG) decreased (*P* < 0.05) (Fig. 3b). However, the overall role of GNG in disposal of the initial glucose load increased over time (see below).

**Fig. 4:**
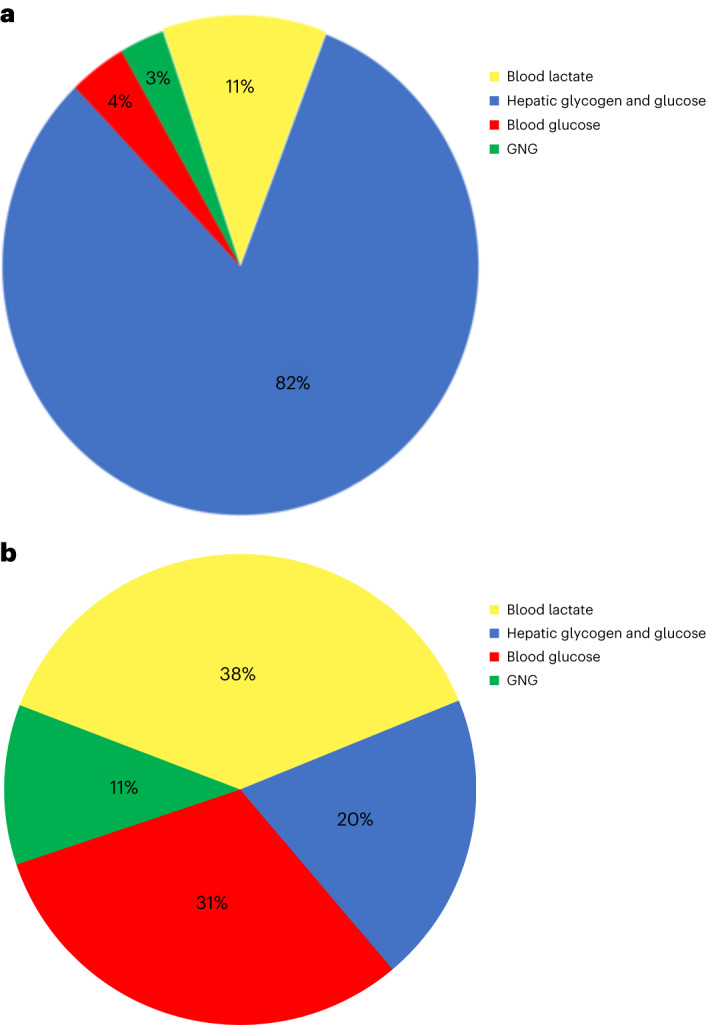
Carbon distribution of hepatic glucose and glycogen, blood lactate and glucose and GNG-derived glucose and the oral glucose challenge. Following an OGTT, most glucose-carbon fluxes through the lactate pool. **a**,**b**, Account of carbon distribution of hepatic glucose and glycogen, blood lactate, blood glucose and GNG-derived glucose 30 min (**a**) and 120 min (**b**) after a 75-g oral glucose challenge. Source data

First-pass glucose carbon retention by the liver after participants ingested glucose solution was calculated by subtracting the combined appearance amounts of lactate, glucose and glucose-derived lactate from the 75-g glucose load. For the first 30-min post-glucose challenge, we determined appearance amounts to be: 9 g as blood lactate, 3 g as blood glucose that bypassed the liver and 2 g as blood glucose derived from lactate via GNG. Hence, the estimate of glucose absorption converted to glycogen or otherwise retained in the liver during the first, enteric PLS phase was 61 g (= 75 −14 g) (Fig. 4a). For the second systemic PLS phase, and overall, we calculated that the 75-g glucose load was accounted for as follows: 29 g as blood lactate, 24 g of glucose from hepatic glucose release, 8 g of glucose from GNG, with 14 g remaining as hepatic glycogen storage or otherwise unaccounted for (Fig. 4b).

To evaluate the veracity of our estimate of the liver’s role in sequestering first-pass dietary glucose uptake, we compared our results to those of Stender et al. who used ^13^C-magnetic resonance spectroscopy (MRS) technology to determine hepatic glucose uptake after a ^13^C-spiked OGTT^12^. Coinciding with our proposed enteric PLS phase, the data of Stender et al.^12^ indicate that hepatic glucose sequestration was detected as soon as 2 min after the oral glucose challenge, peaked at approximately 25 min and declined thereafter.

We used relatively non-invasive means involving glucose and lactate tracers to interrogate the hypothesis of a PLS in healthy young men and women. We observed that responses of arterialized blood [lactate] and appearance rates preceded corresponding changes in [glucose] and furthermore that secondary, incremental changes in blood [lactate] and Ra coincided with those of glucose. Furthermore, for both men and women, we found that the rise in arterial lactate Ra was greater than that of glucose after an oral glucose challenge. Moreover, we found that [insulin] rose before changes in arterialized blood [glucose]. Therefore, we deduce the following: (1) the PLS comprises two components, a first, fast enteric phase of lactate production from gut glycolysis that is followed by a prolonged systemic phase as glucose is disposed of via glycolysis throughout the body corpus; (2) postprandial lactate shuttling is a significant means of managing a dietary carbohydrate carbon challenge; and (3) as arterial insulin rises before glucose, there is also an enteric phase to the insulin response.

We have hypothesized the presence of a PLS^5^ and found evidence for enteric and systemic shuttle phases. Furthermore, we have tested the hypothesis by sampling arterialized blood and measuring lactate and glucose concentrations and kinetics during an OGTT using primed, continuous vascular infusions of d-d_2_-glucose and [^13^C]lactate. This combination of tracers also allowed us to determine GNG from lactate. Most importantly, after an oral glucose challenge we saw an immediate rise in both blood lactate Ra and hence in concentrations. Although there was rise in blood glucose Ra 15 min after the glucose challenge, there was a greater change in lactate kinetics than in glucose (Fig. 3c), indicative of rapid enteric glycolytic activity.

To evaluate the veracity of our estimate of the liver’s role in sequestering first-pass dietary glucose uptake, we compared our results with those of Stender et al. who used ^13^C-MRS technology to determine hepatic glucose uptake after a ^13^C-spiked OGTT^12^. Coinciding with our proposed enteric PLS phase, the data of Stender et al.^12^ indicated that hepatic glucose sequestration could be detected as soon as 2 min after the oral glucose challenge, peaked at approximately 25 min and declined thereafter. Stender et al.^12^ estimated that first-pass glucose sequestration by the liver accounted for ‘most’ of the glucose load; our estimate was 82 ± 2% sequestration (Fig. 4a), the difference attributable to enteric glycolysis producing lactate or glucose that escaped first-pass hepatic sequestration.

In making our calculations we assumed that hepatic glycogenolysis was minimal during the first, enteric PLS phase, but increased later during the subsequent systemic PLS phase. That assumption is justified based on the results of Stender et al.^12^ who showed that initial hepatic glucose sequestration, followed by a continuous decline in liver glycogen, presumably gave rise to the blood glucose appearance explaining the continuous rise in glucose Ra during the systemic PLS phase (Figs. 1c and 2a).

In some respects our data on blood lactate flux are consistent with those of Schlicker et al.^13^. However, they made no attempt to account for the liver’s role in managing carbon flux. Schlicker and colleagues observed uniformly labelled lactate in blood of men after either a mixed meal or OGTT containing [U-^13^C]glucose. Unfortunately, with regard to the presence of an enteric PLS phase, they did not report blood data soon after delivering the glucose challenge (for example, at 5 min). Moreover, it is unclear where blood sampling occurred; an antecubital venous, as opposed to an arterial sampling site, could be assumed. In addition, without a primed CI, they could not calculate lactate Ra or quantitatively account for distribution of ^13^C in the glucose challenge. Despite these limitations, they showed the presence of systemic lactate shuttling as a metabolic buffer to manage a postprandial carbohydrate load.

Our calculations indicate that, on first portal vein blood pass, the liver sequestered most (82%) of the oral glucose load and that only a small percentage of glucose bypassed the liver and appeared in the blood in the first minute after challenge. However, the delayed increase in glucose Ra over time was subsequently disposed of by conversion to lactate, as illustrated by the continuous systemic phase increase in lactate appearance. In accord with our results Gerich and colleagues also observed that glycolysis accounted for most (two-thirds) of the postprandial glucose disposal and that the majority of post-meal hepatic glycogen synthesis in fasted humans was formed via the direct pathway^14^.

The results of our resting, pre-OGTT values of lactate appearance agree well with our previous estimates on healthy young men (1.8–2.6 mg kg^−1^ min^−1^)^15–18^. In the absence of portal vein catheterization, it is problematic to estimate how much lactate from the glucose load contributed to the initial rise in blood [lactate] and Ra. But, from Fig. 3b, it appears that the rates of appearance of blood lactate increased approximately 0.5 mg kg^−1^ min^−1^: if so, approximately 2% of the glucose load appeared as lactate within 5 min (Fig. 4a). Thereafter, the increase in lactate Ra over pre-OGTT represented 38% of the 75-g OGTT. Therefore, although the initial spike in the first (enteral) PLS is perhaps a remarkable finding, the majority of lactate production after an oral glucose challenge occurs during the subsequent, second systemic PLS phase.

Compared with arterial [glucose], the rapid rises in blood [lactate], lactate Ra and insulin are most probably attributable to enteric processes. With lactate gaining further attention as a signalling molecule^19^, the present results may have revealed the presence of a neuroenteric signalling mechanism (enterokine) or mechanisms in humans. An effect of lactate stimulating sensory nerves associated with mesenteric lymphatic fluid may be a mechanism explaining the rapid response to lactate. Using a rodent model, Aponte and collaborators obtained results consistent with the idea that increased enteric lactate may help facilitate insulin secretion by acting on substance P-containing nerves^20^. Furthermore, with regard to enteric lactate signalling, the presence of basolateral lactate transporters (that is, monocarboxylate transporter 1 (MCT-1) and -4)^21–23^ may facilitate neuroenteric signalling processes.

It should be noted that carbohydrate partitioning in 12-h postabsorptive, healthy young people raises the arterial L:P from a nominal value of 10 to one of 20. As such, doubling of the circulating L:P represents a significant systemic redox signal^24–26^. Redox communication via the L:P affects redox status in cytosolic and mitochondrial compartments and, thus, signalling in diverse tissues depending on metabolic rate, physiological state and other conditions. Furthermore, beyond redox signalling including sirtuin activation, lactate affects metabolism via allosteric (for example, transforming growth factor-β and lactate receptor HCAR1), protein and histone modifications^25,27^.

And, finally, it has not escaped our notice that the beneficial effect of metformin on glucose levels in people with diabetes might be via its effect on the PLS. Metformin occurs at higher concentrations in enterocytes compared with the plasma, hence increasing intestinal lactate production after a glucose load^28^. It is possible that an unrecognized role of metformin is to buffer a glucose load by diverting carbon flow to lactate.

In the present study we focused on the effect of an OGTT on parameters of lactate flux. For that purpose, tracers were introduced into the systemic circulation via an indwelling venous catheter with ‘arterialized’ blood sampled from a contralateral, warmed hand vein. In retrospect, sampling catheter placement was fortuitous because, from our previous experience, we might not have seen significant rises in systemic blood lactate concentration or Ra had an antecubital sampling site been used. Notwithstanding the interest in assessing the effect in systemic lactate kinetics via an increase in blood [lactate] and decline in isotopic lactate enrichment, an alternative approach would have been to spike the glucose to be consumed with [^13^C]glucose and look for a rise in the systemic [^13^C]lactate signal after the OGTT.

In the study, we employed a standard OGTT that is used experimentally and clinically. Alternatively, we could have ^13^C spiked a standardized meal tolerance test that would be more relevant to the metabolic consequences of nutrition in daily life. Given the numerous combinations of foods consumed in free-living conditions and the substrate–substrate interactions expected, more research on roles of the gut on energy substrate availability and peripheral disposal will be required to understand the role of a PLS in daily life, health and disease.

In our experiments we had no direct measure of hepatic glucose carbon retention after an oral glucose load, but rather estimated the liver’s role from the blood metabolite flux rates determined. In addition, we had no direct measures of muscle glycolysis during the proposed systemic PLS phase. However, ^13^C-MRS technologies are emerging to test the veracity of assumptions.

And, finally, our methodology did not allow for assessment of the role of the kidneys in managing an oral glucose load; the kidneys probably had a role in GNG from lactate.

Although the current aim of the study was meant to illustrate the enteric production of lactate from glucose, we also observed an immediate rise in insulin concentrations. This rise in insulin within the first 30 min supports our estimates of glycogen storage. Moreover, it may be safe to assume that the total amount of lactate produced from enteric glycolysis after the beverage was underestimated because a fraction of it was probably redirected to the liver and gave rise to glycogen. In aggregate, previous results are entirely consistent with the presence of a second, or systemic, phase to the PLS. And, although postprandial metabolism was not studied by Hui et al.^29^, their data support the role of lactate as a major means of distributing corporal carbohydrate carbon^16,30–32^.

Antiquated ideas of peripheral lactate production caused by hypoxia in peripheral muscles must be supplanted, because first glycolysis proceeds to lactate under fully aerobic conditions and second carbohydrates are processed via lactate shuttling. To rephrase, lactate production and signalling are not peripheral events related to exercise and anaerobic glycolysis in working skeletal muscle, but are rather central processes. Lactate is a major vehicle for carbohydrate carbon distribution and metabolism in mammals including humans.

## Methods

### Subjects

The present study was approved by the University of California Berkeley Committee for the Protection of Human Subjects (CPHS no. 2018-08-11312) and conformed to the standards set by the Declaration of Helsinki. Seven men and eight women aged 21–35 years were recruited via posted notice, newspaper advertisement and social network media. Potential participants were interviewed, and received verbal and written information on study purposes and procedures. After giving verbal and written consent, potential participants were screened for metabolic and cardiovascular diseases (Supplementary Table 1). This involved participants completing a health history questionnaire and 3-d food record forms, and undergoing a blood draw for a basic metabolic panel, ECG and pulmonary function assessments, skinfold measurements, physical examination and $\dot{\mathrm{V}}{\mathrm{O}}_{2}$ peak and ventilatory threshold assessments. For dietary controls, subjects provided 3-d food records that were analysed for caloric intake and macronutrient composition (DietAnalysis Plus, v.6.1, ESHA Research). Only individuals providing evidence of standard, balanced food records, having benign medical histories, passing physical and physiological assessments, and cleared for participation by a licensed physician were entered into the study. Participants included in the study had a body mass index in the range ≥18.5 to <30 kg m^−2^ (ref. ^33^), were non-smokers, had a forced expiratory volume in 1 s/forced vital capacity (FEV_1_/FVC) of >70%, were diet and weight stable, and had fasting blood glucose levels <100 mg dl^−1^ and glycated haemoglobin (HbA1c) levels <5.8%. To assess physical fitness, subject screening included a continual, progressive, leg-cycle ergometer test to assess ventilatory threshold (${\mathrm{V}}{\mathrm{T}}$) and maximal oxygen consumption (VO_2_ peak). Subject screening preceded OGTT testing by at least 1 week. Those entered into the study were provided with verbal and written information on freedom to withdraw from the study, as well as contact information on the laboratory manager, principal investigator and physician should any adverse effects of study procedures occur.

### Tracers

As previously, studies involved primed CI of glucose and lactate and a priming dose of [^13^C]bicarbonate^15,31^. Specifically: [6,6-^2^H]glucose (d-d_2_-glucose, M + 2 signal) (labels lost in glycolysis) gives the rate of glycolysis. Priming and CI cocktails were 250 mg and 2 mg min^−1^, respectively. [3-^13^C]Lactate (label lost in the tricarboxylic acid cycle (TCA) or recycles to glucose in liver and kidneys) gives parameters of lactate production, clearance, oxidation (^13^CO_2_) and GNG (via the Cori cycle, that is, [^13^C]glucose from lactate, M + 1 signal). Priming and CI cocktails were 57.5 mg and 2.5 mg min^−1^, respectively. H^13^CO_3_^−^ was used to prime the bicarbonate–carbonic acid pool, with a priming dose of 136 mg. The ^13^C tracers were given as sodium salts. Isotope tracer cocktails, including boluses and CI cocktails, were prepared by Mariner Advanced Pharmacy and Compounding Company.

### Procedures

The day before the OGTT, the subjects were asked to record and maintain their standard dietary pattern and refrain from strenuous physical exercise. Women who participated did so during the mid-follicular phase of their cycle. After a 12-h overnight fast, the participants reported to the lab, rested for 90 min followed by a 120-min OGTT which started at 07:00. For this, the research team arrived at 04:30 to set up while the subject arrived at 05:00 for study preparation. A warmed hand vein for arterialized blood sampling was catheterized and a contralateral arm vein was catheterized for tracer infusion. No adverse effects of catheterization and tracer infusion were reported.

After set-up and catheterization, background blood and breath sampling for VO_2_, VCO_2_, respiratory exchange rate ($=\dot{\mathrm{V}}{\mathrm{CO}}_{2}/\dot{\mathrm{V}}{\mathrm{O}}_{2}$) and ^13^CO_2_ occurred, and arterialized blood was taken for determinations of endogenous isotopic enrichments as well as insulin and counter-regulatory hormone levels. Subsequently, priming boluses of d-d_2_-glucose, [3-^13^C]lactate and H^13^CO_3_^−^ were given and CIs of d-d_2_-glucose and [3-^13^C]lactate commenced. A 90-min isotope equilibration period was allowed with simultaneous blood and breath samples taken at 75 and 90 min of the CI^15,16,31^.

### OGTT

After 90 min of CI, the participants drank a solution a 296-ml (10-oz) solution containing 75 g of d-glucose (Azer Scientific, catalogue no. 10-0-75). Most subjects consumed the drink in 1–2 min. After the drink, a 2-h timer was initiated and arterialized blood and expired air were taken for determinations of isotopic enrichments (IEs) and glucoregulatory hormones at 5, 15, 30, 60, 90 and 120 min after the OGTT.

### Determinations of IEs

IEs of blood metabolites were as described previously^10,15–17,31^. Blood samples were immediately placed in 7% perchloric acid after collection and then centrifuged at 3,000*g* for 10 min at 4 °C. The clear supernatants were then collected and used for analysis. Samples were then neutralized with 2 M KOH and transferred to ion exchange columns that were previously washed with double deionized water (ddH_2_O) through a cation resin (Analytical Grade 50W-X8, 50- to 100-mesh H^+^ resin, BioRad Laboratories) and with ddH_2_O followed by 2 M formic acid through an anion resin (Analytical Grade 1-X8, 100–200 mesh formate resin). Lactate was eluted through the anion column with 2 M formic acid. The samples were then transferred to a 2-ml gas chromatography vial and lyophilized.

For lactate, samples were resuspended in 200 μl of 2,2-dimethoxypropane and transferred to a vial to which 20 μl of 10% HCl in methanol was added. After samples sat at room temperature for 60 min, 50 μl of *N*-propylamine was added. Samples were then heated for 30 min at 100 °C and subsequently dried under a stream of N_2_ gas, resuspended in 200 μl of ethyl acetate, transferred to a gas chromatography–mass spectrometry (GC–MS) vial and dried again under N_2_ gas, resuspended in 20 μl of heptafluorobutyric anhydride, left for 5 min at room temperature to react and dried under N_2_ gas. Finally, the derivatized lactate was resuspended in 50 μl of ethyl acetate.

For pyruvate, IEs and concentrations were measured via GC–MS of the timethylsilyl-quinoxalinol derivative. As described previously^34^ with slight modifications, 300 μl of perchloric acid extracts were spiked with an internal standard of α-ketovalerate and mixed with 4 M HCl + 4% *o*-phenylenediamine solution (1:1). The solution was heated for 60 min at 90 °C, allowed to cool to room temperature and subsequently extracted using 2.4 ml of methylene chloride. The aqueous layer was removed and the remaining solution was dried under a gentle stream of N_2_. Next, 75 μl of pyridine and 75 μl of *N*,*O*-bis(trimethylsilyl)trifluoroacetamide (BSTFA + 1% trimethylchlorosilane (TMCS)) were mixed and directly added to the dried residue. For alanine IEs, these procedures were repeated to obtain *N*,*O*-bis(trimethylsilyl)alanine.

For glucose, 200 μl of whole blood was placed in 400 µl of ethanol and spun at 10,000*g* for 1 min. A liquid-to-liquid ethanol extraction was preformed and the organic layer containing glucose was extracted and transferred to a 2-ml glass vial and dried under N_2_ gas. The dried glucose was derivatized using 100 μl of a 2:1 mixture of acetic anhydride and pyridine. The GC vial was sealed and heated at 60 °C for 20 min. After 20 min, the sample was dried under N_2_ then reconstituted in 100–500 μl of ethyl acetate.

Lactate LEs were determined by GC–MS (GC model 6890 series and MS model 5973N, Agilent Technologies). Methane was used for chemical ionization with selected ion monitoring of mass-to-charge ratios (*m*/*z*) 328 (non-labelled lactate) and 329 (M + 1 isotopomer, [3-^13^C]lactate). Whole-blood lactate concentrations were determined enzymatically^35^.

Pyruvate LEs were determined by GC–MS on a DB-1701 column of 30 m × 0.25 μm × 0.25 m. Methane was used for chemical ionization with selected ion monitoring of *m*/*z* 233 (non-labelled pyruvate), 234 (M + 1 isotopomer, [3-^13^C]pyruvate) and 261 (α-ketovalerate). Similarly, samples were reinjected and analysed for alanine IEs utilizing 219 (non-labelled alanine) and 220 (M + 1 isotopomer, [3-^13^C]alanine).

Glucose LEs were determined by GC–MS utilizing positive chemical isolation and selected ion monitoring on a DB-17 GC column. The *m*/*z* ratios of 331 (non-labelled glucose), 332 (M + 1 isotopomer, [1-^13^C]glucose) and 333 (M + 2 isotopomer, d-d_2_-glucose) were monitored for the glucose penta-acetate derivative. Whole-blood glucose concentrations were determined enzymatically^36^. Selected ion abundances were compared against external standard curves for calculation of the IEs.

The expired air samples were stored at room temperature until analysed via isotope ratio MS by the University of California, Davis Stable Isotope Facility (Davis, CA).

### Calculations

Lactate and glucose flux rates, that is, rate of appearance (Ra, mg kg^−1^ min^−1^), rate of disposal (Rd, mg kg^−1^ min^−1^) and metabolic clearance rate (MCR, ml kg^−1^ min^−1^) were calculated from the equations of Steele modified for use with stable isotopes^37^:

1 $${\rm{Ra}}=\frac{F-V\left(\frac{{C}_{1}+{C}_{2}}{2}\right)\left(\frac{{{{\mathrm{IE}}}}_{2}-{{{\mathrm{IE}}}}_{1}}{{t}_{2}-{t}_{1}}\right)}{\left(\frac{{{{\mathrm{IE}}}}_{1}+{{{\mathrm{IE}}}}_{2}}{2}\right)}$$

2 $${\rm{Rd}}={{\mathrm{Ra}}}-V\left(\frac{{C}_{2}-{C}_{1}}{{t}_{2}-{t}_{1}}\right)$$

3 $$\% \, {\rm{lactate}}\,{\rm{Rd}}\,{\rm{oxidized}}=\frac{({{\rm{IECO}}}_{2}\times {\dot{\rm{V}}{\rm{CO}}}_{2}\times 90.08)}{(F\times k\times 22.4)}\times 100$$

4 $${\rm{Total}}\,{\rm{lactate}}\,{{{R}}}_{{\rm{ox}}}=\frac{({\rm{Lactate}}\,{{\rm{R}}}{{\rm{d}}}\times \% \,{\rm{lactate}}\,{{\rm{R}}}{{\rm{d}}}\,{\rm{oxidized}})}{100}$$

where *F* represents isotope infusion rate (mg kg^−1^ min^−1^), *V* is the volume of distribution for glucose and lactate (180 ml kg^−1^), *C*_1_ and *C*_2_ are concentrations (mg l^−1^) at sampling times *t*_1_ and *t*_2_, respectively, and IE_1_ and IE_2_ are the excess IEs of lactate at these sampling times. IECO_2_ is the excess IE of expired ^13^CO_2_, $\dot{\mathrm{V}}{\mathrm{CO}}_{2}$ is in l min^−1^, 90.08 is the molecular mass of [3-^13^C]lactate, *F* is the [3-^13^C]lactate infusion rate in mg kg^−1^ min^−1^, *k* is the correction factor for the retention of CO_2_ in body pools, as determined previously^38^ to be 0.83 at rest and 22.4 is the molar volume of CO_2_, a non-ideal gas under standard temperature and pressure conditions.

The percentage of glucose Ra from lactate-derived GNG (%GNG) was calculated as previously described^39^. This approach was derived from that of Zilversmit et al.^40^:

5 $$\begin{array}{l}{\rm{Rate}}\; {\rm{of}}\; {\rm{lactate}}\; {\rm{conversion}}\; {\rm{to}}\; {\rm{glucose}}\\={\rm{GNG}}=\frac{\left(\right.{\rm{Lactate}}\; {\rm{Ra}}\; \times\,({\rm{Glucose}}\; {\rm{M}}+1{\rm{IE}})}{({\rm{IE}}{\rm{Lac}})}\times H\end{array}$$

where glucose M + 1IE is the IE of the M + 1 glucose isotopomer, lactate IE is the IE of lactate and *H* is the Hetenyi factor to correct for loss of label in the TCA during GNG (1.45 at rest and 1.0 during the OGTT^32,41^). The rate of lactate conversion to glucose was calculated as previously described^42–45^ where the M + 1IE is the IE of the M + 1 glucose isotopomer, lactate IE is the IE of lactate and *H* is the Hetenyi factor.

6 $${\rm{Glucose}}\,{\rm{production}}\,{\rm{from}}\,{\rm{lactate}}\,( \% )={\rm{fGNG}}=\frac{({\rm{GNG}})}{({\rm{Glucose}}\,{\rm{Ra}})}\times 100$$

### Estimation of glucose-to-lactate conversion

Subjects were 12-h fasted and consequently GNG and hepatic glycogenolysis were assumed to be active in supporting blood glucose before the oral glucose challenge. Hence, for estimation of glucose-to-lactate conversion after the OGTT, the 75- to 90-min tracer equilibration period before the OGTT was taken as baseline. Furthermore, it was assumed that, after the OGTT, hepatic glycogenolysis was suppressed such that the insulin concentrations rose and lactate Ra equalled the Glu-to-Lac conversion. Values for enteric and systemic PLS phases and total Glu → Lac appear in Fig. 4a,b.

7 $${\rm{Glu}}\rightarrow{\rm{Lac}}\,({\rm{g}})={\rm{Lac}}\,{\rm{Ra}}$$

### Estimations of direct glucose-to-hepatic glycogen conversion

Estimations were based on the assumption that hepatic glycogenolysis was diminished for the first 30 min after the glucose challenge. This assumption was based on Fig. 3a,b as well as the report of Stender et al. using MRS technology^12^. Hence, direct hepatic glycogen synthesis in grams and percentage of the glucose load conversion to hepatic glycogen were computed as:

8 $$\begin{array}{l}{\rm{Enteric}}\,(30\,\min )\,{\rm{hepatic}}\,{\rm{glycogen}}\,{\rm{content}}\,({\rm{g}})\\=75 - [\left(\right.{\rm{Lactate}}\,{\rm{Ra}}\,({\rm{g}})+{\rm{Glucose}}\,{\rm{Ra}}\,({\rm{g}})\,+{\rm{GNG}}\,({\rm{g}})]\end{array}$$

9 $$\begin{array}{l}2\mbox{-}{\rm{h}}\,{\rm{hepatic}}\,{\rm{glycogen}}\,{\rm{content}}\,({\rm{g}})\\=75-[\left(\right.{\rm{Lactate}}\,{\rm{Ra}}\,({\rm{g}})+{\rm{Glucose}}\,{\rm{Ra}}\,({\rm{g}})+{\rm{GNG}}\,({\rm{g}})]\end{array}$$

### Determinations of insulin and glucagon

Blood samples for the determination of plasma hormone concentrations were collected in tubes containing EDTA and protease inhibitors (aprotinin and a dipeptidyl peptidase 4 inhibitor). Concentrations of plasma insulin and glucagon were determined using commercially available ELISA kits (ALPCO).

### Statistical analyses

Analyses were performed using GraphPad Prism v.3.0 software. Group sample sizes were predicted based on power analyses using results of our previous studies^16,30–32^. For comparisons across multiple time points, two-tailed, one-way analysis of variance (ANOVA) was employed, with corrections for repeated comparisons using Dunnett’s tests. For comparisons at two single time points, a two-tailed, paired Student’s *t*-test was applied. The statistical significance for group differences was determined at *α* = 0.05. Data collection and analysis were performed blind to subject identities, but not to sex.

### Reporting summary

Further information on research design is available in the Nature Portfolio Reporting Summary linked to this article.

### Supplementary information

Supplementary Information Participant characteristics.

Reporting Summary

Supplementary Table 1 Source data for participant characteristics.

### Source data

Source Data Fig. 1 Statistical source data for Fig. 1.

Source Data Fig. 2 Statistical source data for Fig. 2.

Source Data Fig. 3 Statistical source data for Fig. 3.

Source Data Fig. 4 Statistical source data for Fig. 4.

Source Data Extended Data Fig. 1 Statistical source data for Extended Data Fig. 1.

## Data Availability

Source data for Figs. 1a–d, 2a–c, 3a,b, 4a,b, Supplementary Table 1 and Extended Data Fig. 1a–c are provided with this paper.
