# Supplementary material for: Enteric and systemic postprandial lactate shuttle phases and dietary carbohydrate carbon flow in humans
Source: Nat Metab. 2024 Feb 22;6(4):670–7. doi: 10.1038/s42255-024-00993-1 (PMC11052717; doi:10.1038/s42255-024-00993-1)
Supplement: Supplementary file 1 — Participant characteristics. [file 42255_2024_993_MOESM1_ESM.pdf]

nature metabolism

Letter

https://doi.org/10.1038/s42255-024-00993-1

Enteric and systemic postprandial lactate shuttle phases and dietary carbohydrate carbon flow in humans

In the format provided by the authors and unedited

Supplementary Table 1. Participant Characteristics

Variable	Men	Women
**Age, yr**	28.1 ± 1.4	25.4 ± 1.8
**Body mass (kg)***	75.4 ± 3.4	59.2 ± 1.7*
**Body Mass Index**	25.1 ± 1.3	21.9 ± 0.7
**Body fat, %**	12.7 ± 1.2	19.7 ± 0.02
**FEV_1/FVC, %**	81.6 ± 3.2	87.1± 7.6
**Absolute VO_2peak ($l \cdot min^{-1}$)**	3.5 ± 0.3	2.2 ± 0.3*
**Relative VO_2peak, ($ml \cdot kg^{-1} \cdot min^{-1}$)**	46.8 ± 3.8	35.5 ± 4.4*
**Peak Power Output, W**	325 ± 20	210 ± 30*
**VT1, % VO_2peak**	77 ± 3	78 ± 2
**3-Day diet records**		
**Energy, $kcal \cdot day^{-1}$**	2914 ± 193	2278 ± 81*
**Carbohydrate, %**	61± 4	61 ± 3
**Fat, %**	30 ± 2	28 ± 1
**Protein, %**	9 ± 1	11 ± 1
**Glucose Load ($g \cdot kg^{-1}$)**	1.0 ± 0.05	1.3 ± 0.04*
**HOMA-IR**	0.61 ± 0.08	1.0 ± 0.1*
**Δf_H 5 Min Post-Challenge (bpm)**	1±2	2±1

Data are mean ± SD; n = 7 males and 8 females; FEV_1, forced expiratory volume in 1s; FVC, forced vital capacity; VO_2peak, peak oxygen consumption; Peak Power Output, Watts (W); VT1, ventilatory threshold 1; HOMA-IR, homeostatic model assessment of insulin resistance; HOMA-%β [45-47]. Δf_H, Change in Heart Rate.* Significantly different between Men and Women ($p < 0.05$).
